# *In Silico* Isomerization Produces Apt Negative Data for VHTS Validation

**DOI:** 10.1021/acs.jcim.6c01764

**Published:** 2026-07-23

**Authors:** Stefan M. Ivanov

**Affiliations:** † Faculty of Pharmacy, 58789 Medical University of Sofia, Dunav 2 Str., Sofia 1000, Bulgaria; ‡ Centre of Excellence in Informatics and Information and Communication Technologies, Sofia 1113, Bulgaria; § Institute of Biophysics and Biomedical Engineering, Bulgarian Academy of Sciences, Acad. G. Bonchev Str., Sofia 1113, Bulgaria

## Abstract

Early on in the emergence of virtual high-throughput screening (VHTS), it was recognized that, for validation to be robust and reliable, decoys should match actives as closely as possible in as many aspects as possible. This has given rise to several generations of validation sets that address previously reported shortcomings of earlier collections. This is an iterative and expensive method of curating validation sets that leaves ample scope for discrepancies between actives and decoys to creep in. It has previously been conjectured that *in silico* isomerization offers an attractive alternative for generating decoys for drug-like compounds that naturally mitigates many of these discrepancies. Here, we explore this proposition and prove the conjecture. We show that isomerization can produce molecules that have hydrogen bond acceptor, donor, rotatable bonds counts, charge, and surface area distributions that match experimental actives more closely than experimental decoys. While these are properties that receive a lot of attention in drug design, we also show that isomerization can effectively produce decoys that are positioned more closely to actives in property hyperspace than current experimental decoys, which tend to be highly dissimilar from the actives. The latter is a significant shortcoming that has thus far remained unreported and unaddressed. Additionally, we introduce the concept of pseudoisomers – decoys that have nearly identical atomic compositions to the actives – and show how they too can be used in VHTS validation at practically no cost. Herein, we build upon the methods, tools, and work of others to facilitate the generation of new and better validation sets more cheaply and efficiently, in the hope of moving the field of VHTS forward toward maturity. To that end, we make our code fully and freely available on GitHub (https://github.com/sivanovMU-Sofia/isomerization).

## Graphical abstract

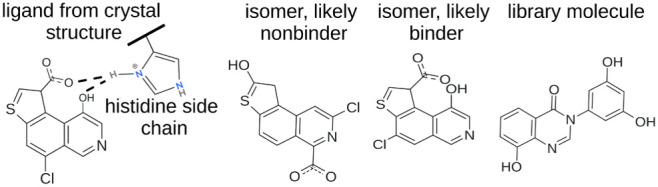

## Introduction

Developing new drugs costs hundreds of millions to billions of dollars^1,2^ with no shortage of diseases and conditions in need of new and better pharmacotherapeutic solutions. Perhaps the most salient example of such a disease is Alzheimer’s where despite decades of research, the underlying etiology remains unclear^3–5^ and the prevalence and socioeconomic burden of the condition continue to rise.^6^ The mounting costs of drug development have prompted many researchers in academia and industry alike to turn to *in silico* modeling and design to reduce the costs associated with preclinical development and to improve the odds of success in clinical trials and postregistration marketing and clinical practice.^7^ This has given rise to computer-aided drug design (CADD^8^) and computer-aided molecular design (CAMD^9^). In the field of hit discovery, the shift from wet lab testing to *in silico* modeling has resulted in the emergence and proliferation of virtual high-throughput screening (VHTS) methods and pipelines. Although nearly all of these use a funnel-shaped model, where increasingly more costly and sophisticated computational chemistry techniques are applied to a progressively decreasing pool of prospective candidates, there is a great variety of alternatives to choose from with no single method, pipeline or product standing out as definitively better or superior to the rest. Therefore, before undertaking a hit discovery project, practitioners must validate the pipelines at their disposal against sets of targets and known binders and decoys. Often, the work involves choosing between two or more products or pipelines from competing vendors or optimizing a given pipeline. To this end, practitioners have compiled validation data sets comprising experimentally confirmed binders against well-known targets of interest. While all sets include experimentally corroborated binders as their positive data, they differ somewhat in how they source their negative data – the nonbinders (decoys). Some require experimental validation for the negatives just like for the positives.^10,11^ Others rely on the low random binding rates and forego experimental measurement on the prospective decoys, opting instead to select ligands from chemical libraries that have been property-matched to the actives.^12–14^ Over time, such sets have gained considerable popularity and have become instrumental in VHTS pipeline development and validation. Of these, some of the most popular ones are the directory of useful decoys (DUD^13^), directory of useful decoys – enhanced (DUD-E^15^), the maximum unbiased validation (MUV^11^) set, demanding evaluation kits for objective *in silico* screening (DEKOIS^16^), and LIT-PCBA.^10^

Very early on, it was recognized that for truly robust and reliable validation, decoys should match actives as closely as possible in as many properties as possible. This is especially true for conceptually simple molecular properties such as molecular weight, numbers of hydrogen bond acceptors (HBA), donors (HBD), and rotatable bonds (henceforth also referred to as rotors), molecular charge, water–octanol partition coefficient (logP), polar and nonpolar surface area which, while simple, are crucial in determining activity. For a method to be proven to be effective at finding actives in a truly prospective setting, it must consistently recognize actives among inactives that are 100 or more times more abundant (which is a typical scenario in prospective screens) and which closely match the actives in properties. The inverse scenario, where there is a significant discrepancy between actives and inactives, hidden or otherwise, often leads to a false and deceptively inflated performance in benchmarking studies. The simplest case is when decoys have much lower weights than actives. In such a scenario, even a null model which does nothing but randomly picking high-weight molecules might appear highly successful. In a prospective screen against a large library of drug-like compounds, however, it would not be able to discern actives from comparably sized or larger decoys and its performance would be reduced to about the random hit rate for the particular target and ligand deck at hand.

This, in turn, has prompted CADD and CAMD practitioners to closely examine the aforementioned validation sets and look for biases. For example, it has been noted that the actives in DUD tend to exhibit a few dominant scaffolds which constitutes analog bias^17^ and that actives and decoys tend to have different net charges.^18^ This has prompted the DUD curators to increase the number of targets, diversify their actives, and attempt to match decoys to actives more closely in terms of molecular weight, estimated logP, numbers of hydrogen bond acceptors, donors, rotatable bonds, and net charge, producing DUD-E.^15^ Despite their commendable efforts, deficiencies and shortcomings remain nonetheless. For example, differences in polar surface area and HBD counts have been noted.^19^ In addition, actives in DUD-E exhibit higher potency than what is observed in prospective high-throughput screens.^10^ Other sets are also not devoid of shortcomings.^20,21^ This brief overview is by no means an exhaustive list of biases and problematic features in current validation sets, many of which likely remain undetected and unreported. Furthermore, this is not a criticism of the authors being cited who have performed important foundational work in the field of computer-aided drug discovery. Rather, this serves to illustrate the difficulty of finding suitable, well-matched decoys even when considering only a handful of “simple” properties such as weight, HBA, HBD, rotors, charge, polar and nonpolar surface area. Further still, while practitioners can keep track of the distributions for a limited number of simple molecular properties, drug-like molecules are highly complex chemical entities that require much more than a handful of features to be thoroughly described. This necessitates using a comprehensive metric to describe and compare molecules and sets of molecules.

Previously, we conjectured that *in silico* isomerization offers an effective and attractive approach to generating decoys for drug-like compounds that naturally mitigates and minimizes the discrepancies between actives and decoys all the while requiring little or no experimental effort and expenditure.^22^ Here, we explore this proposition at greater depth to prove the central premise. In addition, we outline a method to rigorously compare and evaluate data sets in terms of quality. We consider what it would mean for a negative set to be superior to another, how one would go about rigorously proving this, and obtaining such a set through purely computational means. In doing so, we begin by noting that isomerization guarantees a perfect match between an active molecule and its isomer decoys in molecular weight by virtue of preserving atomic composition. Additionally, one could also specifically select decoys with the same number of rotatable bonds and hydrogen bond acceptors and donors. A small margin of tolerance could be introduced, for example ±1 or 2, depending on how hard the search is. At this point, the user is in a good position to generate negative sets that are highly similar to the active ligands; the user already has a set of decoy molecules with the same weight, and closely matching numbers of rotors, HBA, and HBD as the ligands in the active set(s).

Although weight, charge, rotors, HBA, and HBD are important aspects that receive a lot of attention in drug design, drug-like molecules have many more properties that are relevant to their biological activity. Thus, it is typical to represent molecules as multidimensional vectors which encode the presence/absence of features as bit vectors. Complex compounds can then be thought of as data points in a high-dimensional property hyperspace. In this way, a mathematical measure of similarity between two molecules can be produced by computing their cosine similarity, i.e., the cosine of the angle between the two multidimensional vectors. This is obtained by dividing the dot product between the two vectors by the product of their normalized magnitudes. The cosine similarity is 1 if the patterns the vectors encode are identical (i.e., the two vectors represent the same molecule or at least two molecules that match in every property that the vectors encode), 0 if the vectors are orthogonal (i.e., there are no features in common between the molecules that are being compared), −1 if the patterns are inverted, and anywhere between −1 and 1 depending on how similar the molecules are. Once an initial set of decoys is selected by matching weight, rotors, HBA, and HBD, the selection can be further narrowed down. Given a reference data set – MUV, DUD, DUD-E, etc. – all cosine similarities between every active and every decoy are calculated. Then, the density distribution of the cosine similarities is plotted. For the isomerizations, decoys that have higher similarities to the actives are selected. We now have a rigorous, cheminformatics metric for similarity between a set of actives and decoys and a rigorous, cheminformatics metric of how much “better” or “superior” one set is compared to another. For set A to be superior to another set B in terms of decoys, the decoys in A have to be more similar to the actives in A than the decoys in B are similar to the actives in B. Conversely, in an ideal scenario, the actives would be as diverse, i.e., as dissimilar from each other as possible; the same would also apply to the decoys. Using 20 targets from DUD-E and their respective actives and decoys, we demonstrate that isomerization can be used to efficiently generate decoy sets for drug-like molecules that are competitive or even superior to existing decoy sets in terms of quantity and properties such as weight, charge, rotors, HBA, HBD, and molecular surface all the while being appreciably more similar to the actives. Lastly, we compare docking outcomes between DUD-E decoys and isomers. We make our code and analysis tools fully and freely available on GitHub (https://github.com/sivanovMU-Sofia/isomerization) to facilitate future validation efforts and drug discovery projects.

## Methods

1640 actives and 100156 decoys for 20 targets were retrieved in SMILES format from DUD-E for a mean of around 80 actives per target and an average active:inactive ratio of around 1:60. Every active was converted to its raw molecular formula using Open Babel 3.1.1.^23^ Molecular formulas were then fed to MAYGEN^24^ to generate structural (also known as constitutional) isomers in SMILES format. We ran MAYGEN on 4 separate compute nodes equipped with 24 Intel Xeon Gold 5118 2.30 GHz CPUs with multithreading or 96 total CPUs/192 threads. Even for medium-sized drug-like molecules, MAYGEN can often generate upward of 500,000,000 (500 million) isomers in 24 h on a single node. Therefore, for each active, MAYGEN runtime was capped at 10 min during which for most actives millions to tens of millions of isomers to choose from were generated. Subsequently, for each active, we searched for decoys that have a similar number of HBA, HBD, and rotors within a margin of 1 (i.e., ±1). In cases where searching failed to produce at least 100 such isomers, the search criteria were relaxed to ±3. For every active in the set, the search for decoys was initially capped at a time limit of 2 h. Throughout our searches, we excluded isomers with consecutive double bonds in rings (which are unphysical), triple bonds in rings (which are highly strained and unstable structures), and peroxide groups (−O–O−) which are highly reactive and unstable, particularly when multiple consecutive oxygen atoms are chained together. Subsequently, we repeated the search by adding one more constraint – no molecules with spirocycles – and a longer time limit – 4 h instead of 2. The search for suitable isomers was conducted on 4 nodes using all CPUs in parallel (as opposed to consecutively using a single CPU at a time) with the python *joblib* library. For every molecule in the positive set, we ranked its isomer decoys by Tanimoto similarities computed from 2048-bit molecular fingerprints and selected the 100 highest-scoring molecules. For these 100 isomers, we also computed molar masses, polar surface area (TPSA^25^), Labute’s approximate surface area (LabuteASA^26^), and the sums of Gasteiger atomic charges. We calculated and compared the Tanimoto similarities between the actives and the experimental decoys, as well as between the actives and their isomer decoys, i.e., the DUD-E actives – DUD-E decoys similarities and the DUD-E actives – isomer decoy similarities. We also computed and compared the Tanimoto similarities in between the actives, the experimental decoys, and the isomer decoys, i.e., the DUD-E actives – DUD-E actives similarities, the DUD-E decoy – DUD-E decoy similarities, and the isomer decoy – isomer decoy similarities. To obtain a clearer representation of the property similarities and relationships between actives, decoys, and isomers, we projected their respective bit vectors onto a shared 2D space with UMAP (Uniform Manifold Approximation and Projection).^27^ As in previous work,^28^ processing of molecular SMILES representations, computation of chemical properties, and Tanimoto similarities were performed with the open source toolkit RDKit. As before,^29^ we performed two-tailed Mann–Whitney U tests, this time on molecular weights and Tanimoto similarities. Mann–Whitney p-values were calculated with the python *scipy* library.^30^

Lastly, actives, decoys, and isomers were docked to the targets with Smina^31^ and Gnina.^32^ For reference, we also redocked the ligands from the crystal structures into their cognate proteins. For Gnina, we employed an identical docking protocol as in our previous study^22^ using the ligand from the target crystal structure as a reference to define the binding region. With Smina, we used an analogous docking protocol. Different molecules can have the same molecular formula, e.g., CHEMBL460491 (CC1­(CN2C­(=CSC2=N1)­CS/C­(=N\C3CCCCC3)/NC4CCCCC4)­C), CHEMBL508054 (C1CCCC­(CC1)­N/C­(=N/C2CCCCCC2)/SCC3=CSC4=NCCN34), and CHEMBL460298 (C1CCCC­(CCC1)­N/C­(=N/C2CCCCC2)/SCC3=CSC4=NCCN34) all correspond to C21H34N4S2. Therefore, a given isomer molecule can be an isomer of different ligands. We made sure to dock only unique isomers, i.e., duplications were removed. Even so, we have more isomers than DUD-E decoys. Therefore, in order to perform a 1:1 comparison between decoy and isomer docking outcomes, for every target, we selected a subset of isomers equal in number to the decoys. To make the selection criterion unambiguous and uniquely defined, we sorted the isomers by Tanimoto similarities and retained the top N where N is the number of DUD-E decoys for the target.

Before docking, actives, decoys, isomers, and crystallographic ligands were converted from SMILES text format to 3D SDF format with Open Babel 3.1.1. In all cases, actives, decoys, isomers, and crystal ligands were scored by Smina affinity (for Smina docking) and Gnina pose score (for Gnina docking). Ligands were ordered by the respective metrics. The rankings of the actives when using the DUD-E decoys and MAYGEN isomers of the actives as negative controls were analyzed across tools and targets. We also report and analyze the respective top 1% enrichments.

## Results

In the initial search, out of the 1640 actives in the data set, our automated decoy generation and search pipeline successfully produced 100 isomers for 93% of the molecules in the positive set. This compares favorably with the average of ∼60 for the experimental decoys. Overall, our pipeline was successful in producing and identifying isomers that are well matched to their parent actives. As shown in Figure 1A, there is a perfect match between actives and isomerization decoys in terms of molecular weight to the point where the blue and red lines for their respective distributions lie perfectly one over another such that they merge into a single pink line. Only isomerization can provide such a perfect match in molecular weight. As expected, the molecular weight distribution for the experimental decoys closely matches the actives' distribution but does not overlap it perfectly. The p-values are helpful to get an intuitive understanding of the distributions. When dealing with three sets of different sizes, comparing p-values across them as an indication of more or less overlap is valid only when two of the sets overlap perfectly (p = 1) and the other two do not (*p* < 1). This is indeed the case for the molecular weight distributions where actives and their isomer decoys overlap perfectly but actives and experimental decoys do not. For HBA, HDB, rotor counts, charge, TPSA, and LabuteASA, there is less than perfect overlap. Because all three sets have different sizes, we cannot directly compare p-values in between them to infer whether there is more or less overlap between any two pairs. Therefore, in these cases, p-values are not given. Nonetheless, panels B–G in Figure 1 show that there is generally good correspondence between actives, experimental decoys, and isomerization decoys in terms of molecular properties, particularly for total surface area (LabuteASA, Figure 1G) where there is near perfect overlap between actives and isomers and a very good, albeit lesser overlap between actives and experimental decoys. The near perfect overlap between the positive set and the isomer negatives for total surface area is because atomic composition is preserved during isomer generation. Moreover, we see that there is also very good overlap in terms of polar surface area (TPSA, Figure 1F), albeit less so than with total surface area. This shows that polar surface area changes more significantly during atom rearrangement than total surface area (LabuteASA). It is also notable that there is very good correspondence between actives and isomers in terms of molecular charge (Figure 1E), more so than between actives and experimental decoys where the charge distribution for the experimental decoys exhibits long tails to the left and to the right of the actives' distribution. Conversely, the isomer decoys' distribution closely matches the actives' distribution, particularly in its span. In all likelihood, this is also because isomerization preserves atomic composition. This makes it easier to match molecular charge between actives and isomer decoys than between actives and experimental decoys that are completely unrelated to the actives and can have very different atomic compositions.

**Figure 1:**
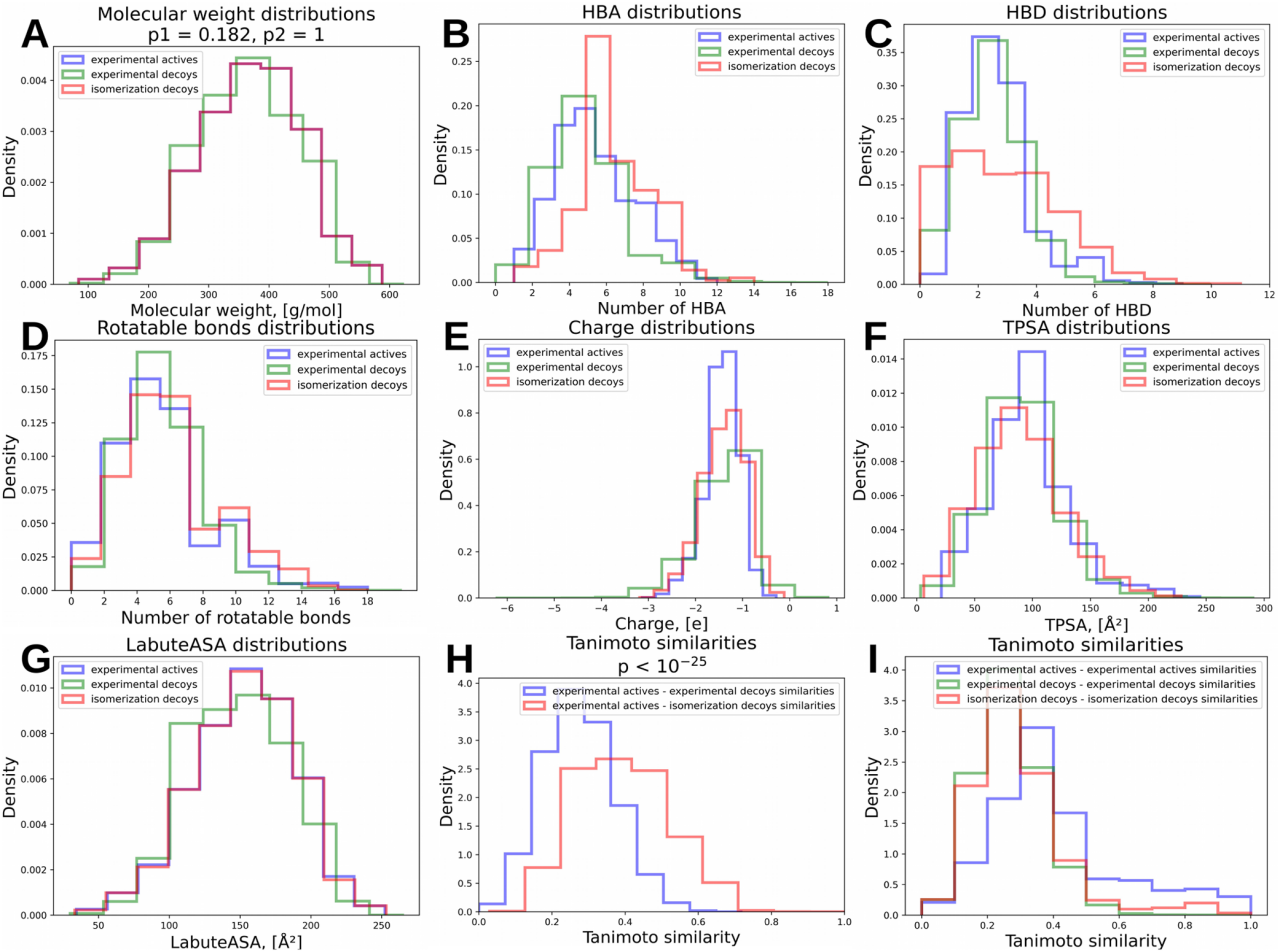
Molecular property distributions for DUD-E actives, DUD-E decoys, and isomer decoys. (A) Molecular weight distributions. P1 is the p-value from the two-tailed Mann–Whitney U test between the molecular weights of the DUD-E actives and DUD-E decoys; p2 is the p-value between the DUD-E actives and isomer decoys. Note that in this panel, the red and blue lines overlap perfectly into a single pink line. (B) Distribution of the number of hydrogen bond acceptors (HBA). (C) Distribution of the number of hydrogen bond donors (HBD). (D) Distribution of the number of rotatable bonds. (E) Molecular charge distributions. (F) Polar surface area (TPSA) distributions. (G) Total surface area (LabuteASA) distributions. (H) Tanimoto similarity distributions between DUD-E actives and DUD-E decoys and DUD-E actives and their isomer decoys. The two-tailed Mann–Whitney U test p-value is also given. (I) Tanimoto similarity distributions in between DUD-E actives, DUD-E decoys, and isomer decoys.

Crucially, we see that the Tanimoto similarity distribution for the isomerization decoys (compared to the actives, Figure 1H) is right-shifted toward 1 compared to the distribution for the experimental decoys. The p-value is below 10^–25^. Such a small value indicates a high degree of statistical significance for the difference between the two distributions. Intuitively, one can think of it as one distribution being different from the other in its positioning on the X axis. Our results show that overall, isomer decoys lie closer to experimental actives than experimental decoys lie to the actives in chemical property hyperspace. Furthermore, we see that there still is a considerable degree of similarity in between the actives – in Figure 1I, a significant fraction of the active – active Tanimotos lie above 0.6 and even above 0.8. In contrast, a much smaller proportion of experimental decoy – experimental decoy and isomer – isomer Tanimotos lie above 0.6.

Expanding the one-dimensional (1D) projection of property similarities to a shared two-dimensional (2D) space with UMAP^27^ reveals far more details about the interrelationships between actives, DUD-E decoys, and isomer decoys and their relative distances in property hyperspace. Figure 2 shows UMAP plots generated from the bit vectors of the actives (blue dots), decoys (green dots), and isomers (red dots) for every individual target in our set. The 2D projections reveal that although our set contains between 40 and 100 actives per target, in reality the actives for most targets occupy no more than a handful of points with multiple ligands lying on top of each other in the projections. It immediately becomes apparent that the active set for every target contains ligands from a single-digit number of chemical series. Indeed, in the case of pur2 and cxcr4, all actives essentially lie on 1 or 2 points; for hxh4 there are a couple more points, but all of them lie relatively close to each other. Further, we see that the DUD-E decoys are generally little better in terms of diversity – in most cases, they cluster closely together in a relatively narrow patch of the plots meaning that they also lie close together in property hyperspace. Conversely, the isomers tend to be much more spread out in the plots which indicates that they are much more diverse than the actives and the decoys.

**Figure 2:**
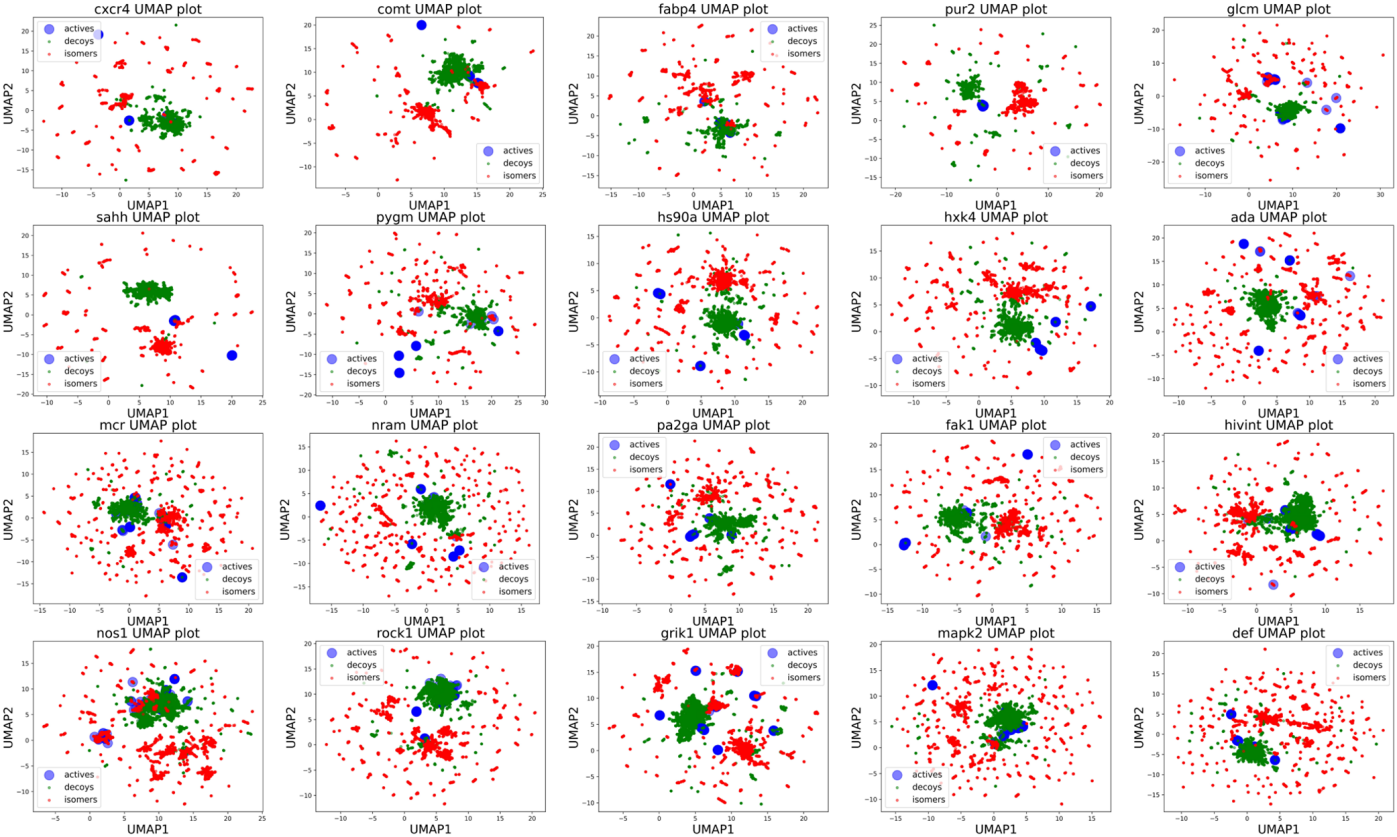
UMAP plots of the actives, decoys, and isomers for every target. The active points are deliberately made larger to become noticeable in the plots.

Figure 1 provides density distribution plots for the chemical properties we examine here for the entire set; breakdowns by target are provided in SI Figures 1–9. Examining the detailed breakdowns by targets reveals that results for the individual targets are consistent with the patterns we have described for the entire set. Molecular weights are perfectly matched between actives and isomer decoys (SI Figure 1), followed by total surface area (SI Figure 7). More often than not, the isomer distributions are more tightly centered around the experimental actives' distributions than the experimental decoy distributions. For certain targets and properties, the experimental decoys form a long tail that spans far beyond the actives. This is the case, for example, with the charge distributions for pur2 and pygm (SI Figure 5) or the HBD distribution for cxcr4 (SI Figure 3). In other cases, the experimental decoys' distribution is significantly shifted away from the actives' distribution, e.g., molecular weight for pur2 (SI Figure 1); HBA counts for cxcr4, sahh, and hsa90 (SI Figure 2); HBD counts for comt and pur2 (SI Figure 3); charge distributions for cxcr4, comt, and fabp4 (SI Figure 5). Crucially, for all 20 targets, the isomer decoy Tanimotos are right-shifted compared to the experimental decoy Tanimotos (SI Figure 8). This confirms that overall, isomer decoys are more similar to their parent actives than the experimental decoys, i.e., actives and isomers lie more closely to each other in property hyperspace than actives and DUD-E decoys. Moreover, we see that there is a considerable degree of similarity in between the actives (SI Figure 9) where in most cases, the active – active Tanimoto distributions are right-shifted compared to the experimental decoy – experimental decoy distributions. Generally, isomer – isomer distributions follow the active – active distributions more closely than the experimental decoy – experimental decoy distributions.

We now examine the docking results and compare outcomes and performance between Smina and Gnina across targets. Docking all ligands and ordering them by score produces a monotonic score vs rank function; marking the positions of the actives produces a plot known as a recovery plot^22^ In an ordered list of scores, a hypothetical perfect predictor would place all binders at the beginning of the list; a perfectly random estimator would space out actives evenly throughout the list, i.e., the X axis of the recovery plot. When there is a large number of actives whose rankings need to be tracked, recovery plots can become visually cluttered. In such a scenario, it becomes more convenient to transform the actives’ rankings into violin plots.^22^ Figure 3 shows the violin plots for every target. For a tool to perform better than another, its distribution should be more highly concentrated near 1 (the beginning of the list) and have a lower-lying median (the white dashes in the violin plots); its interquartile range (the thick black bars in the plots) should be smaller and lower-lying. Figure 3 shows that neither tool (Smina or Gnina) definitively outperforms the other. In certain cases, Gnina outperforms Smina, e.g., pur2; in others, the roles are reversed, e.g., fabp4. For certain targets, e.g., nram, the actives’ rankings are better (i.e., lower) with the decoys than the isomers which means that the isomers are more challenging as negatives for the docking tool. For most of the targets here, however, actives score better when isomers are used as negative controls. Moreover, there does not seem to be a general, consistent rule ordering performance between Smina and Gnina, decoys and isomers. For certain targets, one tool performs better on the isomers than the decoys (e.g., Smina, comt); for others, it performs better on the decoys (e.g., Smina, mapk2). In the case of sahh, the order of performance is Smina isomers > Smina decoys > Gnina isomers > Gnina decoys (best to worst). In other cases, the order is the exact opposite (e.g., def). More often than not, the ordering is some variation in between. This is also reflected in the top 1% enrichments (Figure 4) where there are no absolute winners and losers.

**Figure 3:**
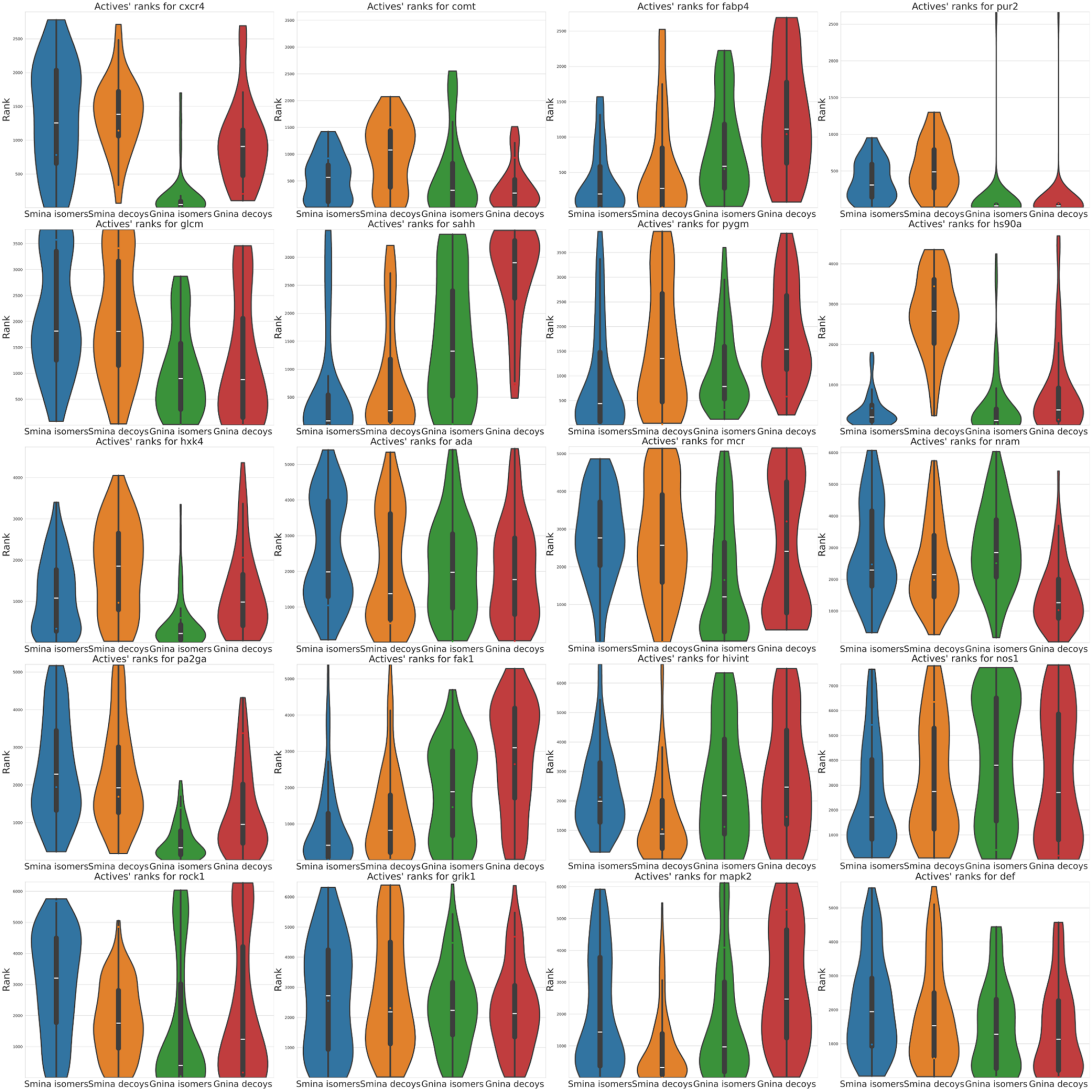
Ranking violin plots for every target. The binding protein–ligand pair ranks when sorting by Smina and Gnina score are plotted. In every panel, the ordering is as follows: actives’ ranks by Smina scores (actives against MAYGEN isomers), actives’ ranks by Smina scores (actives against DUD-E decoys), actives’ ranks by Gnina scores (actives against MAYGEN isomers), actives’ ranks by Gnina scores (actives against DUD-E decoys). The white dashes represent the medians of the distributions; the colored dots inside the black bars represent the ranks of the crystal ligands redocked into the cognate protein structures; the thick black bars are the interquartile ranges; the thin black bars are the remaining data points.

**Figure 4:**
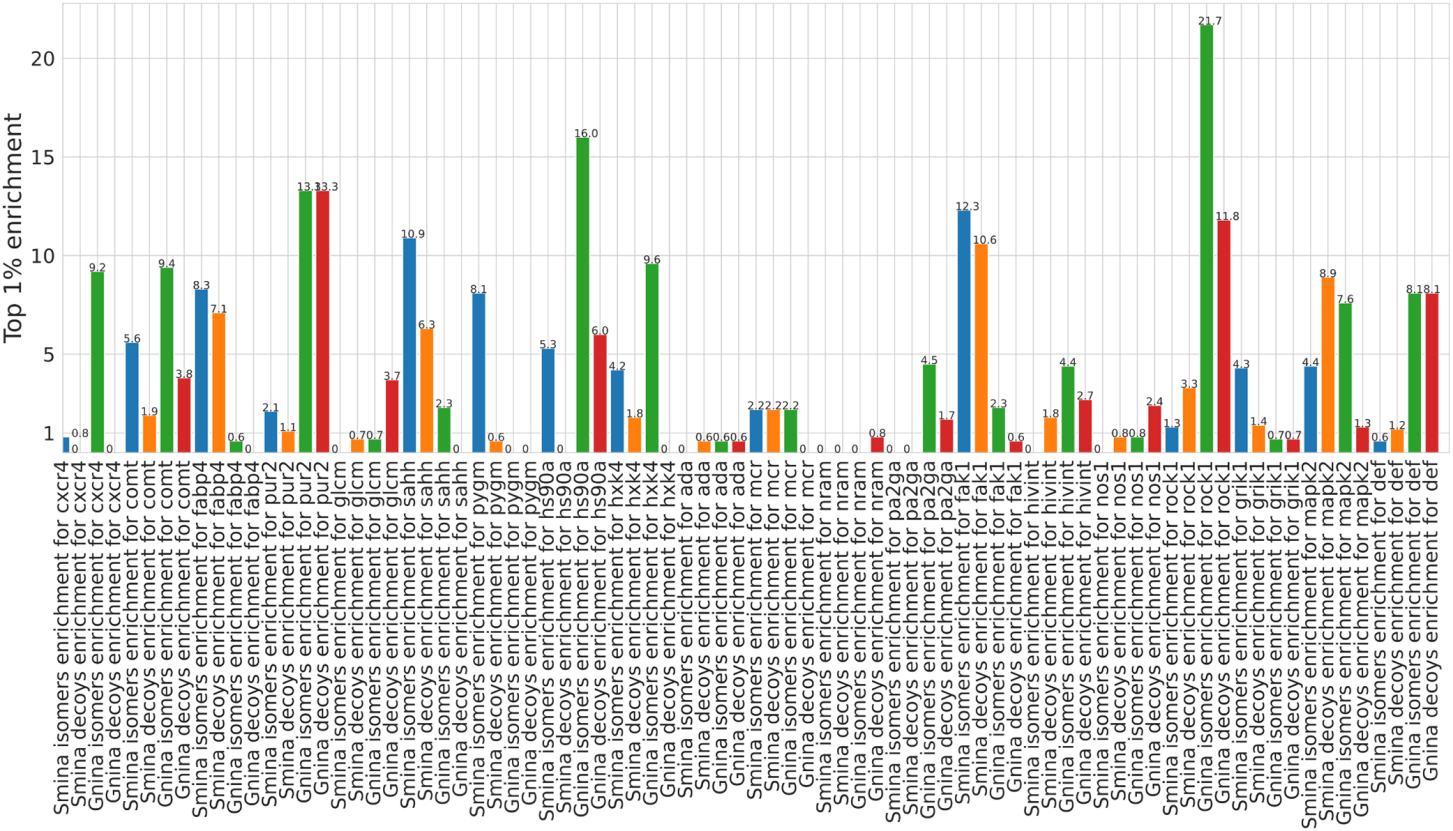
Top 1% enrichments. For every target, the ordering is as follows: enrichment by Smina scores (actives against MAYGEN isomers), enrichment by Smina scores (actives against DUD-E decoys), enrichment by Gnina scores (actives against MAYGEN isomers), enrichment by Gnina scores (actives against DUD-E decoys). The enrichment is the number of actives in the top 1% divided by the number of actives that would be placed in the top 1% by random selection ((number of binders/number of binders + number of nonbinders)/100). The y = 1 line, i.e., an enrichment of 1, corresponds to random selection; enrichments above 1 indicate better than random selection; enrichments below 1 indicate worse than random selection. An enrichment of 0 means that no actives have been placed in the top 1%. Enrichments are also given numerically on top of every bar.

It is notable that for certain targets (e.g., cxcr4, fabp4, pur2) the actives achieve superior rankings with the isomers rather than the decoys meaning that the isomers are less challenging. This is contrary to what might be expected given the superior property matching. This suggests that the isomer sets contain unfavorable chemistries that the docking programs penalize. Inspecting the isomers that were generated revealed that many of the molecules contained large, strained spirocycles. Such molecules scored poorly and were easily outscored and outranked by the actives. Therefore, we retained the original ligand filters and added one more – no spirocycles – and increased the search time from two to four hours. Repeating the docking with the new set of isomers and comparing the actives’ rankings between the two rounds confirms that for many of the targets the new sets become more challenging (Figure 5), e.g., cxcr4, comt, fabp4, hsa90a.

**Figure 5:**
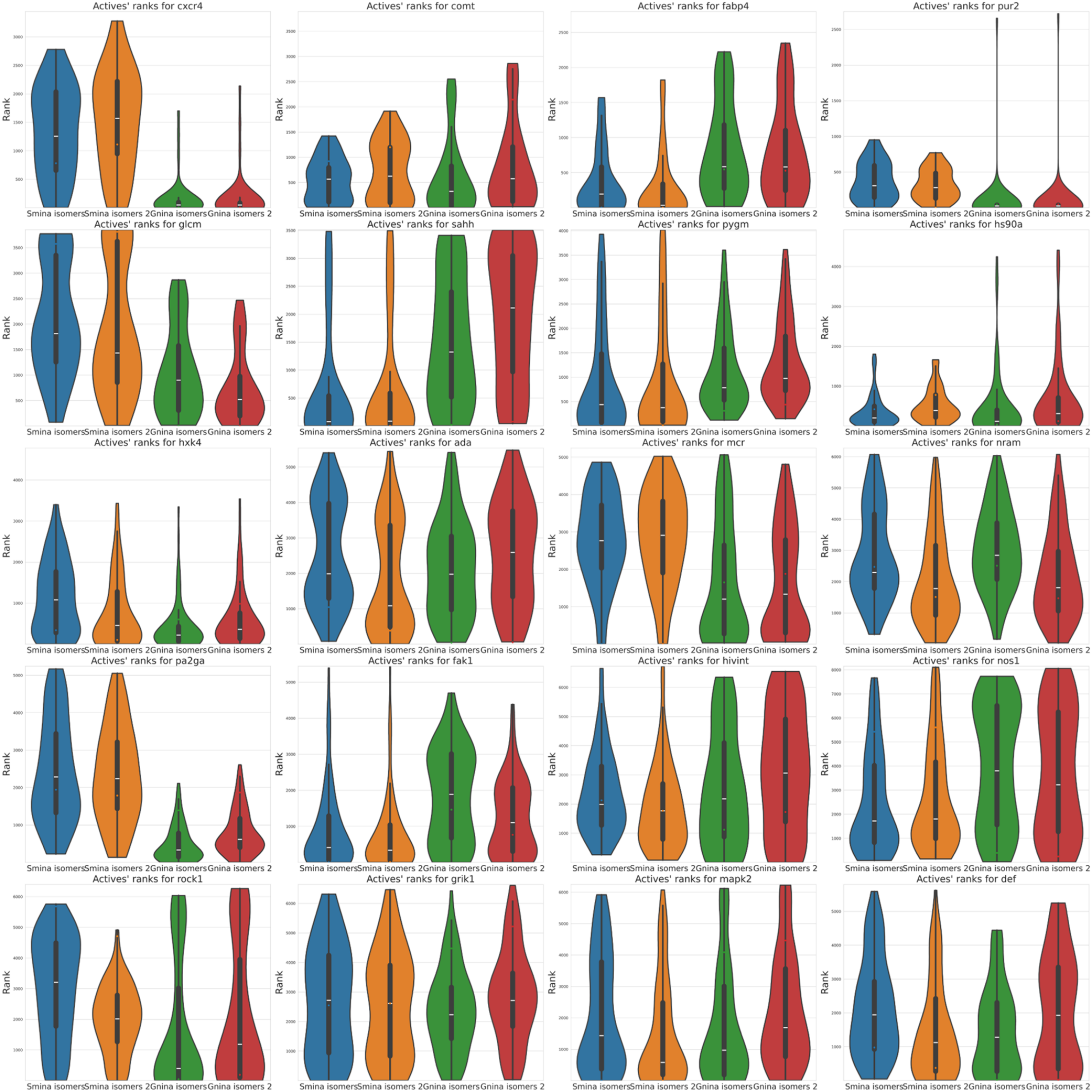
Ranking violin plots for every target. The binding protein–ligand pair ranks when sorting by Smina and Gnina score from the two rounds of isomer searching are plotted. In every panel, the ordering is as follows: actives’ ranks by Smina scores (actives against MAYGEN isomers from the first round), actives’ ranks by Smina scores (actives against MAYGEN isomers from the second round), actives’ ranks by Gnina scores (actives against MAYGEN isomers from the first round), actives’ ranks by Gnina scores (actives against MAYGEN isomers from the second round). The white dashes represent the medians of the distributions; the colored dots inside the black bars represent the ranks of the crystal ligands redocked into the cognate protein structures; the thick black bars are the interquartile ranges; the thin black bars are the remaining data points.

## Discussion

With the present report, we demonstrate that computational isomer generation can successfully be used to generate ample decoy sets that are comparable or superior to experimental decoys in key properties such as HBA, HBD, rotor counts, charge and molecular surface. Crucially, we show that isomer decoys tend to be more similar to active ligands than their experimental decoy counterparts. Remarkably, around half of isomers occupy a sort of “Tanimoto Goldilocks zone” between 0.4 and 0.8 (Figure 1H). Below 0.4, there is little to nothing in common between actives and decoys which might make it easier even for poor models to correctly recognize decoys for simply being “not like the actives.” Conversely, above 0.8, one approaches a region in parameter hyperspace that is close to the actives where the risk of encountering actives (i.e., false negatives) begins to increase. This is evidenced by congener series where in most cases, the Tanimoto similarities between binders and nonbinders are above 0.8.^33–36^ In stark contrast to the isomerization decoys, the vast majority of experimental decoys lie below 0.4 in terms of Tanimoto similarity to the actives (Figure 1H). So far this has remained as a hidden bias. This is highly problematic because it makes it easier for computational chemistry techniques and ML models trained on this set to correctly recognize many of the decoys as such not by any real understanding of intermolecular interaction and association but simply as “not like the actives.” This deceptively increases performance in benchmarking studies using such sets. While we can confirm some of the discrepancies in polar surface area, charge, HBA, and HBD counts and the high degree of similarity between the actives that have been reported before, herein, we report a bias that is likely far more significant. Namely, for many of the targets in published validation sets, decoys are far too dissimilar from actives. This makes training and benchmarking on these sets unrealistic and counterproductive because such models and tools would struggle in prospective drug discovery screens on large ligand libraries where decoys with high similarity to the actives for the target at hand may be present.

Expanding the 1D property projections to 2D with UMAP^27^ has the added advantage of avoiding the time-consuming N × N calculations of pairwise similarities all the while providing much more information than the 1D representations. Therefore, UMAP calculations offer more insight for less computation than Tanimoto similarities.

Herein, we present a general pipeline. From then on, users are encouraged to closely examine their specific system – the target and the molecules that are known to be active against it, and to carefully consider what other properties they might need to select their decoys for. As an example, we will consider the Bcl-2 family antiapoptotic proteins which drive multiple malignancies.^37–48^ Their binding site has been extensively characterized structurally and energetically^28,49^ and has attracted a lot of interest from medicinal chemists looking to develop novel antiproliferative agents. One such prominent series of antiproliferative molecules targeting the Bcls are the benzoylsulphoneamidoaryls^37,50,51^ Benzoylsulfoneamidoaryls bind in an extended conformation and make extensive contacts with the proteins’ NWGR motifs through the sulfonamide moiety.^52^ Consequently, when designing new analogs, practitioners might want to weigh features that correspond to the benzoyl or arylsulfonamide groups in the respective bit vectors more heavily or discard molecules that do not have one or both of these features outright. On the GitHub page accompanying this work (https://github.com/sivanovMU-Sofia/isomerization), we include code that illustrates how chemical features can be encoded as Daylight SMARTS patterns that can then be searched and selected for or against.

We reiterate that any biases and deficiencies in current data sets we uncover and highlight are not criticisms of the respective authors. Instead, they are intended purely as constructive criticism of the sets, not the work and its authors, and serve to illustrate the difficulty of finding diverse actives and suitable decoys, the latter of which is in essence an optimization problem in a highly dimensional hyperspace. This problem is made exponentially more difficult by the constraints imposed by synthetic accessibility. Although certain molecules may be highly suitable to serve as decoys because of excellent matching to actives in properties and a favorable degree of chemical similarity, if they are difficult to synthesize, they will not be found in data sets from screens that require experimental testing of the negatives. Similarly, they will not appear in data sets sourced from commercial chemical libraries because vendors would likely not include compounds that are very difficult to synthesize. With the present report, we demonstrate that *in silico* modeling can fill such gaps left behind by experiment as *in silico* modeling is not hindered by synthetic accessibility. Indeed, decoys do not need to be easy to synthesize to be used for *in silico* modeling and validation; they do not need to be synthesized at all. These are some of the very cost savings and efficiencies that underpin and enable VHTS, CADD and CAMD. Moreover, isomerization by its very nature maintains a direct correspondence between actives and inactives – for every active, the user always knows its corresponding inactives with perfect accuracy. This naturally is highly useful in a VHTS setting where one aims for as much correspondence in chemical properties between positives and negatives as is possible to achieve. This is because maximizing correspondence in positives and negatives requires fine control over the properties of the nonbinders.

To conclude this line of reasoning, we will now consider how molecular properties emerge from atomic composition. Some, by their very nature, are a direct result of atomic composition and are not affected by atomic connectivity. The most prominent example of such a property is molecular weight – once an atomic composition is fixed, so is the molecular weight, irrespective of how the atoms are rearranged. Next come properties which depend more strongly on atomic connectivity. Our work shows that two such properties in increasing order of dependence are total surface area (LabuteASA) and polar surface area (TPSA). *In silico* isomerization, then, is an astute approach to generating negative data because it is a natural “shortcut” through parameter hyperspace to a region closer to the actives which is where CADD practitioners should aim to position their decoys. By maintaining atomic composition fixed, isomerization immediately produces decoys with a perfect match in properties that do not depend on connectivity. As for properties that do, isomerization is nonetheless a shortcut because it is easier to find good matches in a set where atomic composition is preserved than in a set of completely unrelated molecules. This is because an identical atomic composition, even if not a perfect guarantor of property matching, still contributes to maintaining properties in a desired range. In other words, maintaining atomic composition is more likely to produce decoys with properties similar to the actives' because atomic composition is a major determinant of molecular properties. Conversely, it is harder to find suitable decoys from a set of molecules that are completely unrelated to the actives because such molecules would in all likelihood have very different atomic compositions which, again, are a major determinant of molecular properties. Lastly, in the drug-like molecular space, isomerization provides abundant decoys to choose from at practically no cost. Here, we build on the data sets, tools, and efforts of others in the hope of collaboratively moving the field forward toward better validation and more efficient tools for hit discovery. Hopefully, these tools will then deliver the new drugs so very much needed in the 21st century.

When using different sets of molecules for the actives and the inactives, it is inevitable that there will be some discrepancy in one or more chemical properties. The goal in such a scenario should be to *minimize and mitigate* discrepancies while increasing (i.e., improving) the Tanimoto similarities between actives and inactives. A perfect correspondence between actives and decoys can only be achieved by pairing ligands to different proteins some of which bind the actives in question whereas others do not such that the same ligands are present in both the positive and negative set.^22^ For example, one can take protein–ligand pairs from PDBbind.^53,54^ These would be the positive data. Practically unlimited amounts of negative data can be generated by randomizing the ligands across proteins (accounting for cases where a ligand is paired with the same protein it is known to bind but from a different PDB structure). Although this is the only way to achieve a perfect match between actives and inactives (because the actives and the decoys in this case are the exact same set of ligands),^22^ this scenario is much more challenging for computational chemistry because it involves evaluating different ligands paired with different proteins rather than the much more common scenario of evaluating the same protein paired with different ligands. Currently, practitioners tend to make use of the extensive error cancellation when evaluating different ligands with the same protein conformation^55–58^ and even so, VHTS often yields high false positive rates and low hit rates.^59,60^ For now, computational chemistry is simply not ripe enough to be as reliable as experimental measurements.

One might expect Gnina to outperform Smina on every target given that Smina uses an empirical scoring function developed using a small data set and redocking only,^31^ whereas Gnina has a much more modern neural network based pose scorer that was trained and validated on a much larger data set comprising both redocked and crossdocked protein–ligand pairs.^32^ Nonetheless, there are instances where Smina outperforms Gnina in terms of overall recovery and 1% enrichment such as sahh and fak1 (see Figures 3 and 4). Therefore, users should not view rankings and outcomes as absolute. At most, one can try to make a probabilistic argument that one tool outperforms another more often than not. In stark contrast, what is definitively proven here is that *in silic*o isomerization can indeed be incorporated in VHTS pipelines as a viable source of negative data.

Generating useful isomers is particularly simple in cases with a clear, experimentally validated pharmacophore that has been proven to be indispensable for ligand activity. This is illustrated in Figure 6A where we have a hypothetical pharmacophore consisting of a suitably spaced carboxylic and hydroxyl group simultaneously interacting with a positively charged protein side chain, e.g., an arginine or a doubly protonated histidine. In this scenario, one can easily generate nonbinding isomers by separating the hydroxyl group and carboxylate far apart and away from the arginine/histidine. Such ligands would be much better suited for comparisons to the actives than the typical biaryls which are often found in drugs and drug-like molecules^61^ and compound libraries.^62–65^ Interestingly, in such cases, isomerization can likely generate additional binders as well. For example, given the active molecule in Figure 6A, moving around one of the heteroatoms or shifting the position of the chlorine slightly is not likely to abrogate activity, as long as the key pharmacophore remains intact. Generating useful isomers is likely to require little method development effort to become much more efficient. Typically, isomerization tools^66–68^ attempt to generate all possible isomers^24,69^ for a given structure or molecular formula. For drug-like molecules, this often creates a vast search space that has to be traversed to find isomers that have desirable properties and are positioned close enough to the corresponding active in property hyperspace. We believe it would take relatively little effort to develop search algorithms that are specifically designed to produce suitable isomers by simultaneously preserving or minimizing changes in numbers of rotors, HBD, HBA, charge, etc.

**Figure 6:**
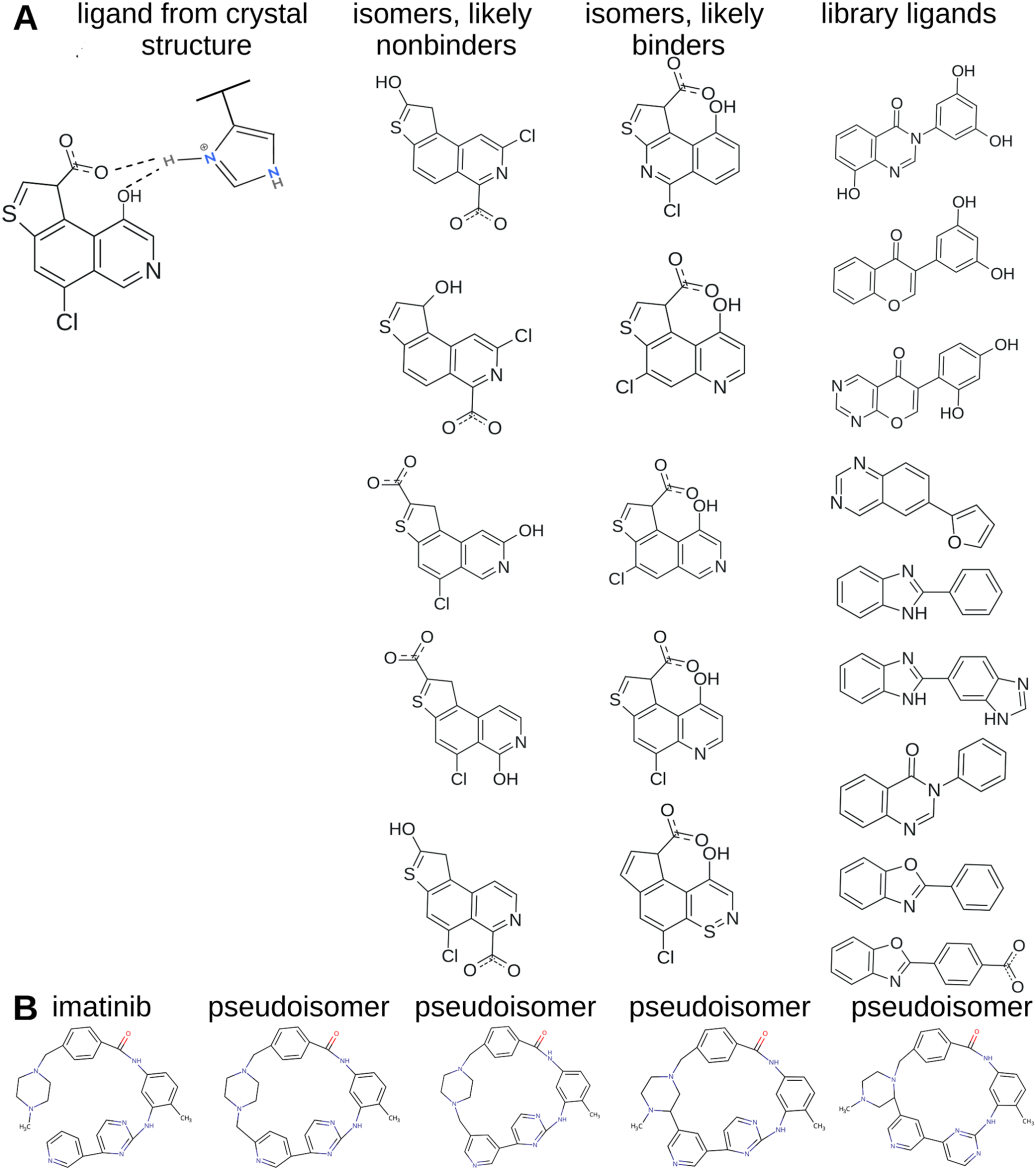
Examples of decoy molecules generated by closely matching known actives. (A) Decoys generated from an experimentally validated pharmacophore observed in crystal or NMR structures. The pharmacophore consists of a suitably positioned carboxylic and hydroxylic group simultaneously interacting with a doubly protonated histidine side chain from a protein. Separating these groups or placing them in locations where they cannot interact with the histidine is likely to produce isomers that are inactive. Conversely, making small changes to the active ligand such as slightly altering the position of a single heteroatom or the chlorine is likely to produce active isomers as long as the key pharmacophore remains intact. Biaryls that can often be found in compound libraries are also shown for comparison. (B) Imatinib and derivative analogs that closely match its atomic composition – pseudoisomers. The pseudoisomers have been generated by closing the molecular structure into a large ring by introducing one more covalent bond. They have an identical heavy atom composition to the parent compound and a highly similar hydrogen content; they differ only by two hydrogen masses because of the additional bond.

For the case in Figure 6A, to generate a large number of suitable isomers, one could simply shuffle around the OH or COO^–^ moieties, heteroatoms, or chlorine rather than generating completely unrelated structures, most of which would not be of use. While this can certainly be done by drawing structures by hand, it is time-consuming, tedious, and error-prone. Clearly, automation is the solution to this problem. We are most hopeful that the method development part of the cheminformatics and computational chemistry community takes an active interest in this proposition.

We show that isomers can be made more difficult as negatives by examining the results and introducing additional filters in an iterative manner. In this way, negative sets of any desired difficulty can be produced given sufficient compute resources. Crucially, we also identify a key vector for future method development to greatly improve efficiency. Rather than generate, output, parse, and analyze millions to billions of molecules only to identify them as undesirable and discard them, it would be vastly more efficient if users could pass a list of undesired SMARTS patterns to the isomerization tool so that it would not output or better yet not generate at all molecules that match these patterns. We believe it would take little effort to modify existing tools in this way. Moreover, this would nicely dovetail into efforts to develop isomerization tools and algorithms that preserve the numbers of rotors, HBA, HBD, etc., given an input molecule.

Further still, one does not need to be constrained by Tanimoto similarity while selecting decoys. Here, we demonstrate it only as an example, an unambiguous criterion to select a subset of molecules from a much larger set. All of the calculations we describe here are CPU-based; they are cheap and can be massively parallelized. In an industrial setting, when the goal is to generate more challenging negative sets and cloud computing^70,71^ is available, after removing undesired chemistries, practitioners need not retain only the top N most similar ligands. Many more molecules could be docked to identify and retain the best scoring negatives. There is a lot to be learned from generating and analyzing such sets – which molecules score well and why?

Finally, isomers are not the only source of negative data; existing chemical libraries could also be filtered for chemical properties that fall within a reference range defined by a set of actives and filtered for undesired chemistries. We recommend isomerization because it maintains atomic composition and therefore facilitates property matching. However, if a user does not want to go through the trouble of generating and analyzing millions to billions of isomers, property matching and filtering could be applied to existing collections rather than isomers.

Structural data on protein–ligand binding for key signaling nodes and targets opens up additional promising avenues for decoy generation. We will examine imatinib as an instructive example. It is an FDA approved drug marketed under the brand name Gleevec (among others) as an oral therapy for various types of cancer.^72^ Imatinib is a small molecule inhibitor targeting multiple kinases.^73,74^ Extensive structural characterization on it and its active analogs^75,76^ has shown that the inhibitor binds the kinase in an extended conformation fitting tightly into a long, narrow, tunnel-like binding site. An intuitive way to generate nonbinding analogs that preserve many of the features and properties of the active parent is to simply introduce one more covalent bond, transforming the long and slender molecule into a bulky ring system that cannot possibly fit into the binding site. In such a scenario, the decoys maintain the heavy atom composition of the active parent; their atomic composition differs only by two hydrogens because of the additional covalent bond. We refer to such analogs as pseudoisomers because, while the atomic composition is not exactly the same, it is highly similar to the parent compound. Such pseudoisomers are illustrated in Figure 6B. Clearly, many more of these decoys can be generated for imatinib and its analogs.^76,77^ Another avenue for producing useful isomers and pseudoisomers would be to fuse multiple rings together such that they too cannot fit in the kinase binding site. Such isomers and pseudoisomers would be placed in a highly strained conformation when docked in the binding site. Clearly, such molecules and structures would be very useful when validating methods to calculate ligand strain.^78–82^ All of the above vectors for future work – both in method development and decoy generation – provide ample scope for many more papers to come by ourselves and hopefully others as well.

## Conclusions

With the current report, we demonstrate that computational isomer generation is cost-effective and successful at generating negative data that is of comparable or even superior quality in terms of simple but crucial molecular properties. Even more importantly, isomerization produces decoys that are positioned closer to the actives in property hyperspace than what experimental searches or screening of chemical libraries typically produce. Further still, isomerization entails maintaining a direct correspondence between actives and decoys. This naturally facilitates fine-tuning the properties and property distributions of the decoys. Isomerization provides a vast search space where isomers can be selected for or against additional desired or undesired properties. Moreover, we expect method and algorithm development in this field to make searches more efficient by producing suitable isomers in a targeted rather than random manner. In addition, we introduce the concept of pseudoisomers – molecules that have nearly the same atomic composition as the actives – and demonstrate how they too can be useful when validating VHTS pipelines and other computational chemistry techniques. Once suitable isomers and pseudoisomers are generated, they are to be run through VHTS pipelines that usually involve molecular docking and dynamics^83–85^ and free energy techniques of varying sophistication.^86–94^ We strongly encourage practitioners to validate every step of their pipeline by examining the ranking of known actives among decoys at every step rather than just the final ranking.^22^ In our experience, steps and techniques which are assumed or believed to be productive often turn out to be unproductive or even counterproductive. When using stochastic tools or methods in VHTS, users should report estimates for the variation in ranking^22^ rather than simply reporting results from a single run that can never be reproduced exactly.

We cannot foresee or anticipate every property of every target and every ligand deck every user is going to encounter; we can only offer general guidelines and good practices. Rather than a fixed data set which is unlikely to perfectly fit the needs of even a single practitioner, we describe and provide a means to tailor negative sets on a case-by-case basis. We deliberately illustrate the ideas presented herein with simple, freely accessible GitHub scripts (https://github.com/sivanovMU-Sofia/isomerization) rather than closed-source software, thereby lowering the barrier to entry as much as possible and broadening the utility of these concepts as much as possible.

Over time, we expect improvements in computational estimates of enthalpy,^95^ solvation,^96^ and entropy^97–100^ that form the binding free energy^101–103^ to translate into improved performance in VHTS screens.^22^ This, in turn, should translate into improvements and cost savings in drug discovery and design. It stands to reason that an *in silico* traversal of conformational hyperspace should reproduce experimental affinities and rankings given a sufficiently mature force field^104,105^ and sufficient sampling. It is currently unknown whether a more efficient method exists to accurately rank ligands by their affinity to a given target. As a loose analogy, we now have multiple algorithms for calculating the value of π that differ greatly in their complexity and computational cost.^106–110^ In stark contrast, we currently know of only one algorithm to obtain the free energy of binding and to correctly rank ligands by that energy which is to traverse the conformational landscape^111,112^ available to every protein–ligand pair.^113^ This is what happens when a protein and ligand come together in a test tube or *in vivo* and what various free energy techniques are trying to emulate. Whether there is a more efficient method of reproducing these with perfect accuracy in currently not known.^114^ This is certainly an interesting proposition, as it would allow further cost savings by “short-circuiting” reality itself. We conclude by noting a curious intersection between drug discovery, computational chemistry, metaphysics, and the simulation hypothesis^115^ – if one can prove that such a short circuit does not exist, i.e., it is energetically, computationally, and financially cheaper to inhabit a reality rather than to simulate one,^116–120^ this would constitute strong, perhaps definitive, evidence against the simulation hypothesis.

## Supplementary Material



## Data Availability

All relevant data are within the manuscript, attached figures, and supplementary files. The entirety of the code used in the preparation of this manuscript is freely available on GitHub (https://github.com/sivanovMU-Sofia/isomerization).

## Notes

Figures 1–9: breakdowns of molecular properties by target (PDF)
