# Supplementary material for: In Silico Isomerization Produces Apt Negative Data for VHTS Validation
Source: J Chem Inf Model. 2026 Jul 23;66(15):9246–61. doi: 10.1021/acs.jcim.6c01764 (PMC13471428; doi:10.1021/acs.jcim.6c01764)
Supplement: Supplementary file 1 [file ci6c01764_si_010.pdf]

In Silico Isomerization Produces Apt Negative Data for VHTS Validation

Stefan M. Ivanov[1,2,3*]

1. Faculty of Pharmacy, Medical University of Sofia, Dunav 2 str., Sofia 1000, Bulgaria
2. Centre of Excellence in Informatics and Information and Communication Technologies, Sofia 1113, Bulgaria
3. Institute of Biophysics and Biomedical Engineering, Bulgarian Academy of Sciences, Acad. G. Bonchev str., Sofia 1113, Bulgaria

***correspondence: sivanov@ddg-pharmfac.net**

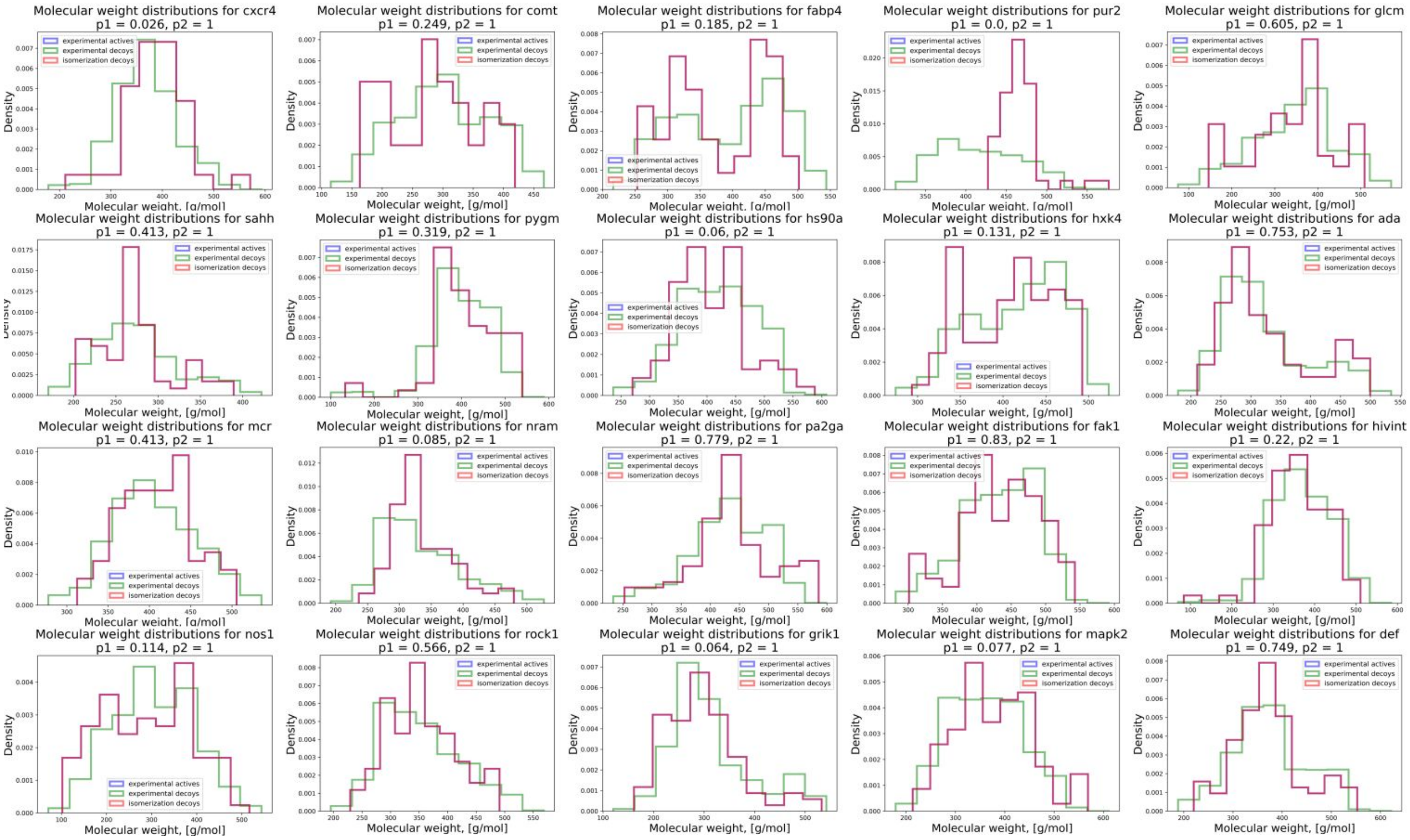

SI Figure 1. Molecular weight distributions for DUD-E actives, DUD-E decoys, and isomer decoys by target. P1 is the p-value from the two-tailed Mann-Whitney U test between the molecular weights of the DUD-E actives and DUD-E decoys; p2 is the p-value between the DUD-E actives and isomer decoys. Note that in all panels, the red and blue lines overlap perfectly into a single pink line.

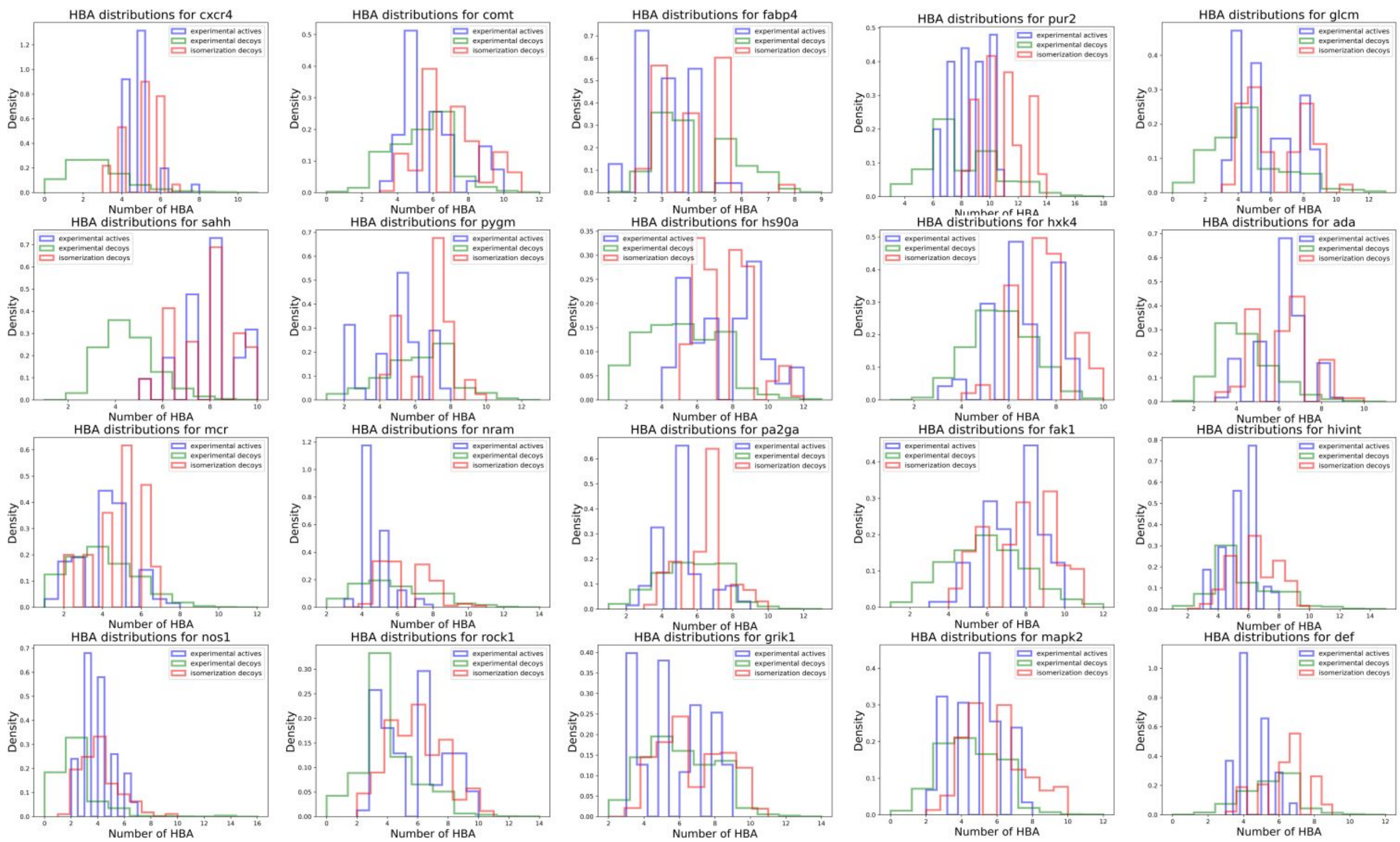

SI Figure 2. Distribution of the number of hydrogen bond acceptors (HBA) by target.

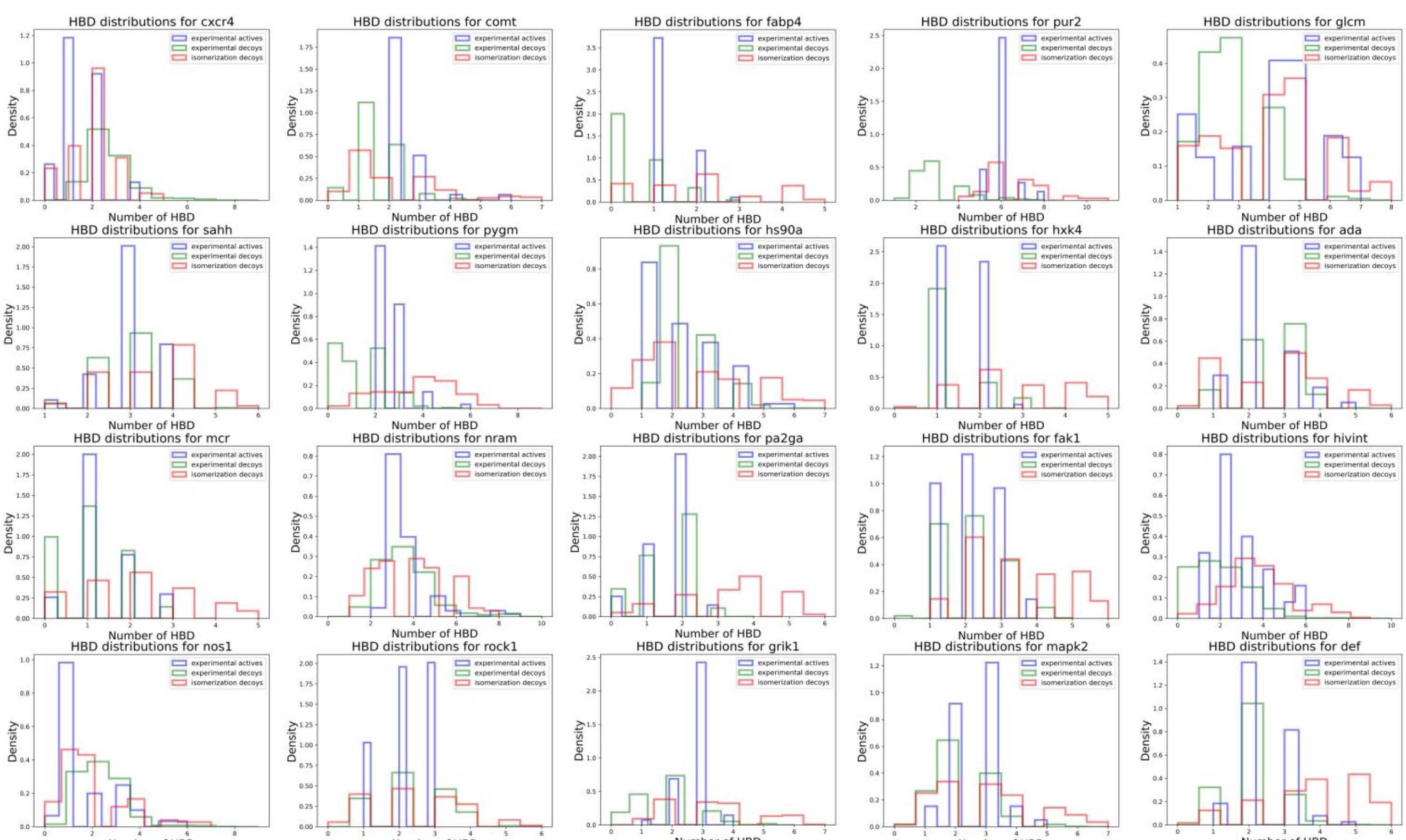

SI Figure 3. Distribution of the number of hydrogen bond donors (HBD) by target.

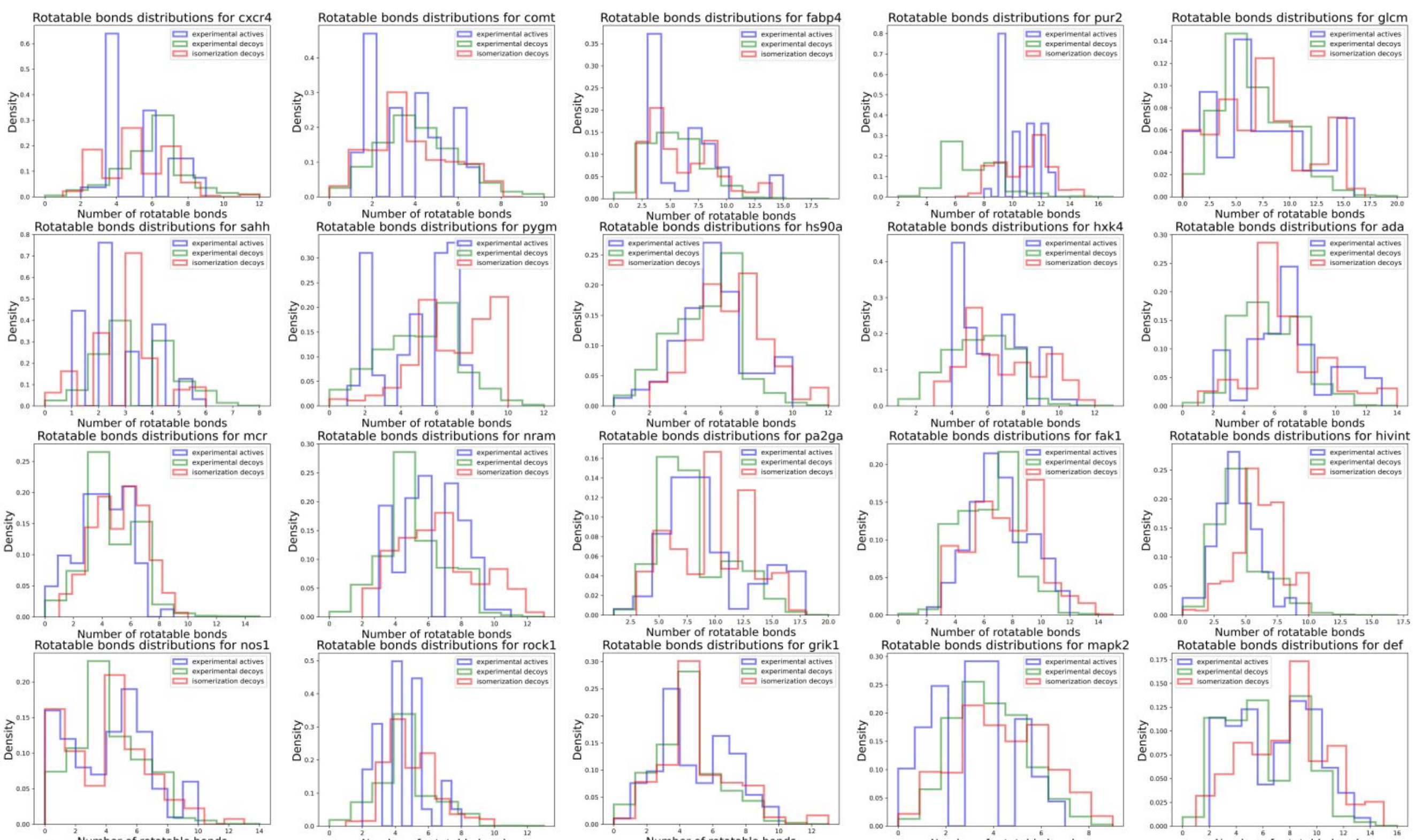

SI Figure 4. Distribution of the number of rotatable bonds by target.

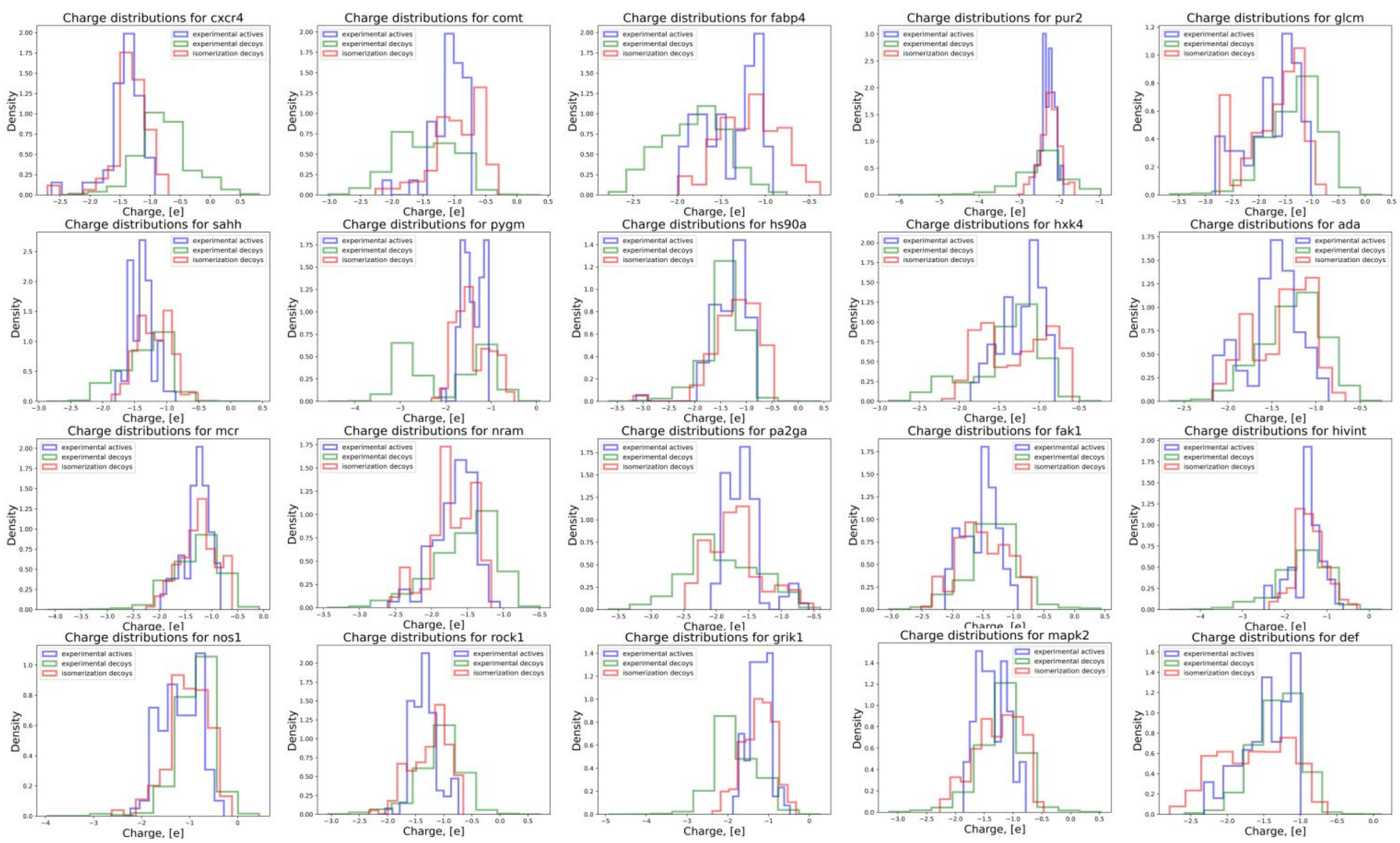

SI Figure 5. Distribution of molecular charge by target.

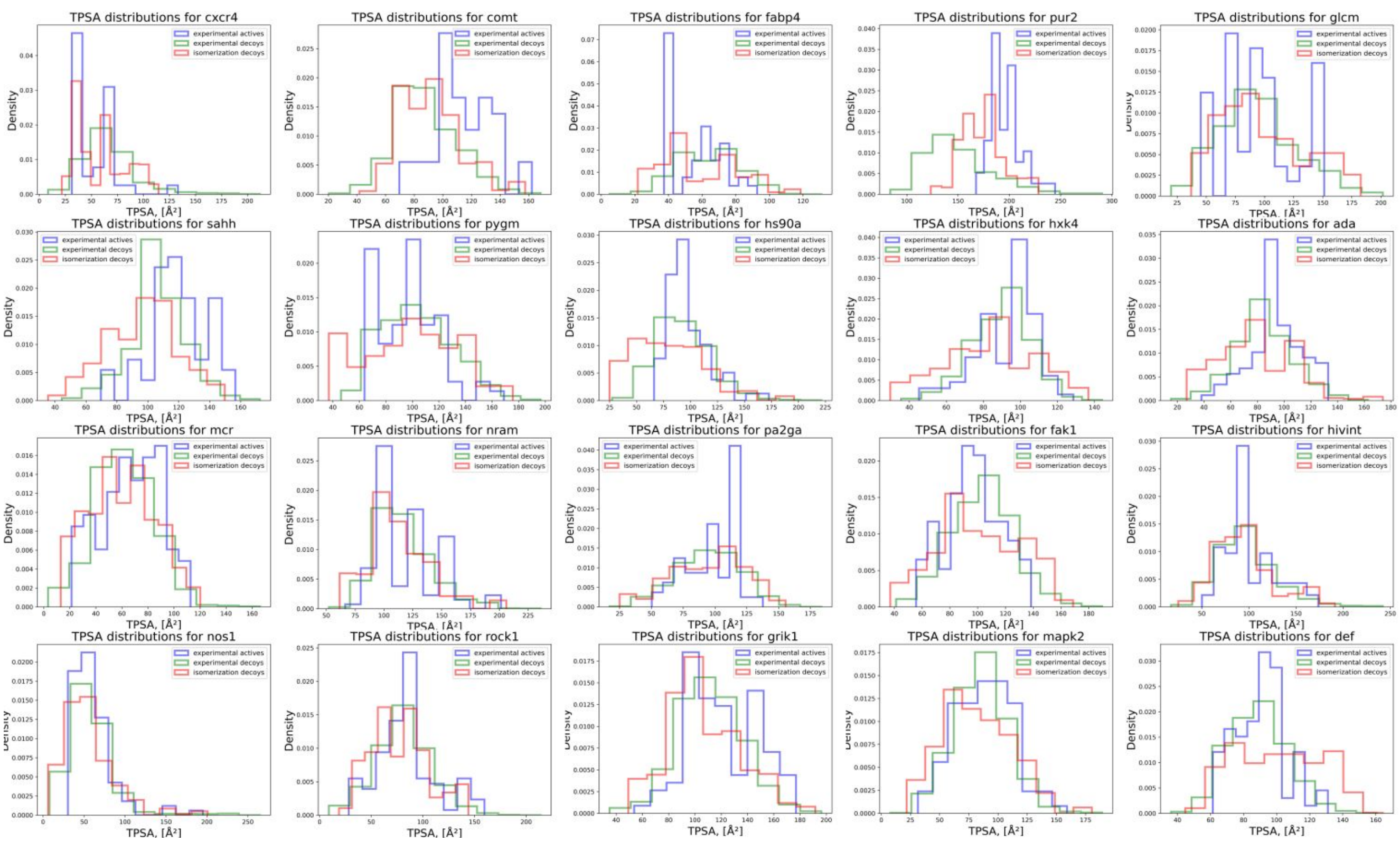

SI Figure 6. Polar surface area (TPSA) distributions by target.

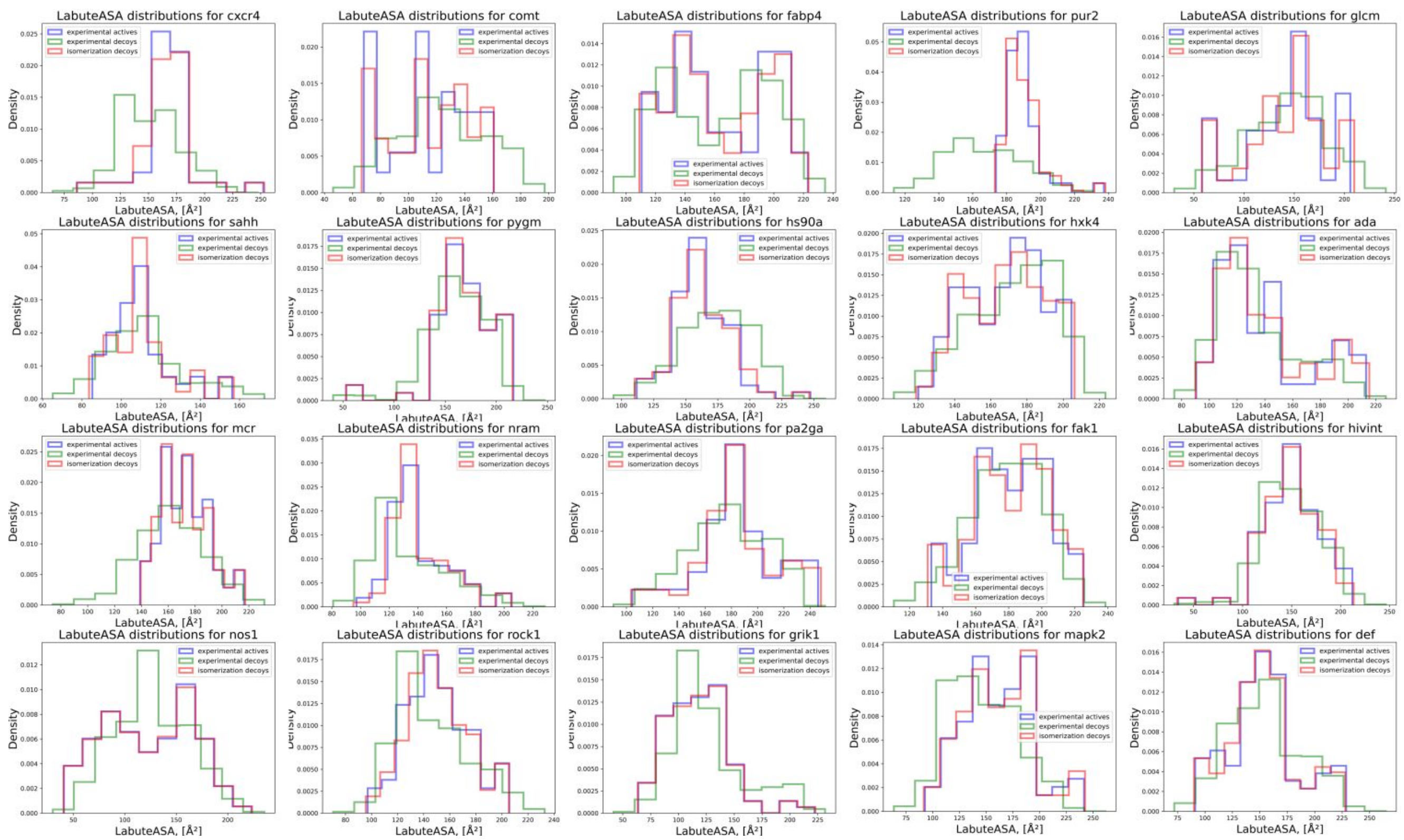

SI Figure 7. Total surface area (LabuteASA) distributions by target.

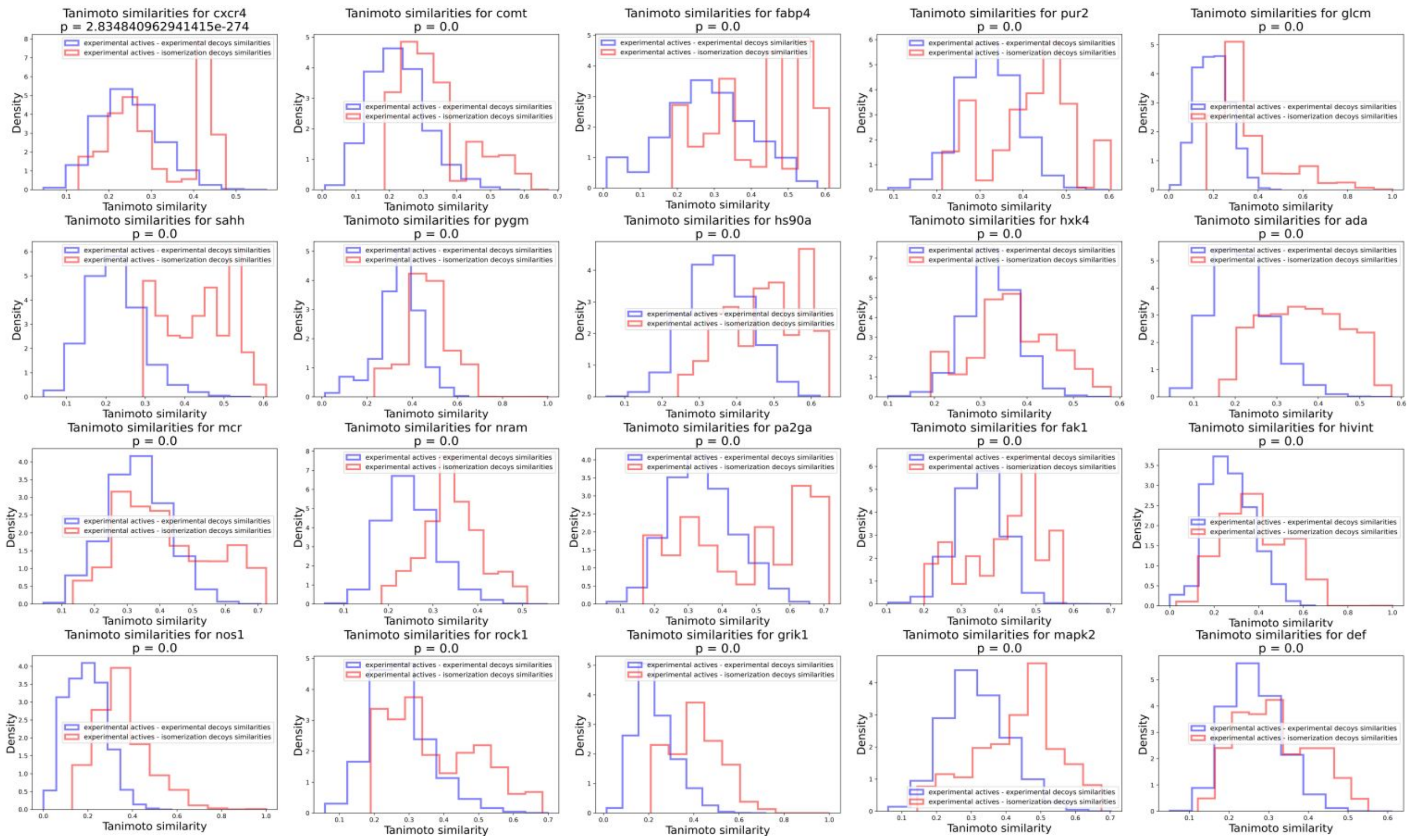

SI Figure 8. Tanimoto similarity distributions between DUD-E actives and DUD-E decoys and DUD-E actives and their isomer decoys by target. The two-tailed Mann-Whitney U test p-value is also given. In most panels the p-value is given as 0.0 because it is too small ($p << 10^{-25}$) to be given precisely.

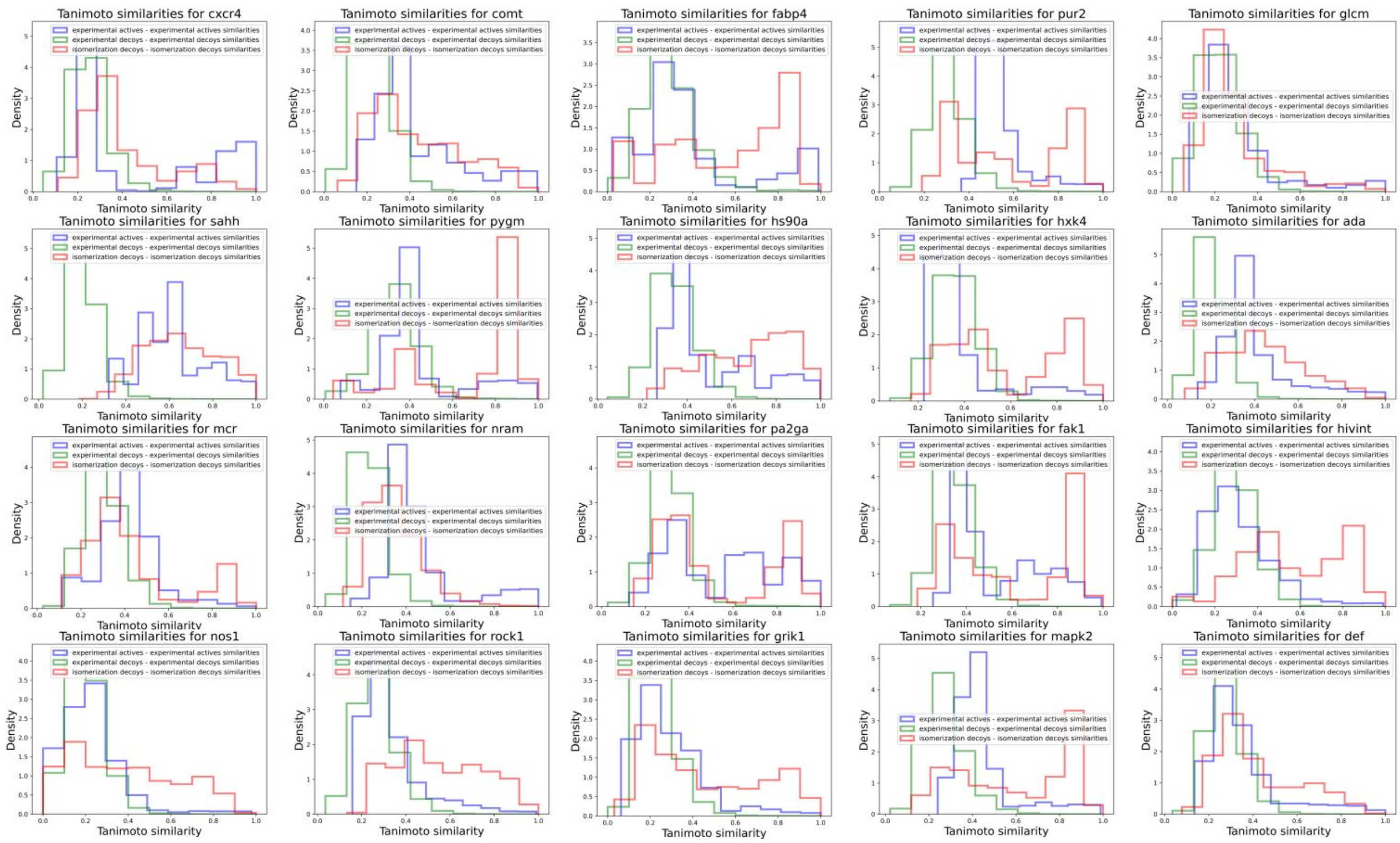

SI Figure 9. Tanimoto similarity distributions in between DUD-E actives, DUD-E decoys, and isomer decoys by target.
